# FastMDAnalysis: Software for Automated Analysis of Molecular Dynamics Trajectories

**DOI:** 10.1002/jcc.70350

**Published:** 2026-03-29

**Authors:** Adekunle Aina, Derrick Kwan

**Affiliations:** ^1^ Department of Physics California State University Dominguez Hills Carson California USA; ^2^ Biophysics Program California State University Dominguez Hills Carson California USA; ^3^ AAI Research Lab California State University Dominguez Hills Carson California USA

**Keywords:** molecular dynamics, reproducibility, trajectory analysis, workflow automation

## Abstract

The analysis of molecular dynamics (MD) trajectories remains fragmented, requiring researchers to integrate multiple computational methods in bespoke scripts. This creates a significant barrier to reproducibility and limits analytical scope. We present FastMDAnalysis, a unified framework that establishes a reproducible, automated workflow for end‐to‐end trajectory analysis. The system orchestrates a comprehensive and extensible suite of core analysis modules, including root‐mean‐square deviation and fluctuation, radius of gyration, hydrogen bonding, solvent‐accessible surface area, secondary structure assignment, dimensionality reduction, clustering, fraction of native contacts for protein folding studies, and dihedral angle analysis, within a single, consistent environment built on MDTraj, scikit‐learn, and SciPy. The software natively supports all major trajectory formats, including GROMACS, AMBER, and CHARMM. We demonstrate a >90% reduction in code volume for standard workflows and validate its numerical equivalence to reference implementations. FastMDAnalysis provides a methodological advance that makes rigorous, multi‐analysis MD studies accessible and reproducible for the computational chemistry, biology, and biophysics communities. The software is freely available under the MIT license at https://github.com/aai‐research‐lab/fastmdanalysis.

## Graphical abstract

FastMDAnalysis is a unified, automated framework that transforms complex, fragmented molecular dynamics analysis into a single, reproducible command. It integrates essential biophysical analysis, reducing scripting effort by >90% while ensuring full numerical accuracy for rigorous, publication‐ready insights.

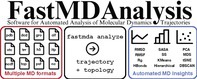

## 1 Introduction

The analysis of molecular dynamics (MD) trajectories remains a major bottleneck in computational chemistry, motivating the development of FastMDAnalysis, a software framework that standardizes and automates end‐to‐end trajectory analysis while preserving the numerical accuracy of established libraries. MD simulation has evolved into an indispensable tool in computational chemistry, biophysics, and materials science, providing atomistic insights into the structure, dynamics, and function of biomolecular systems and nanomaterials [1, 2]. Driven by advancements in force field accuracy, sophisticated sampling algorithms, and modern high‐performance computing architectures, the temporal and spatial scales accessible to MD have expanded dramatically, now routinely reaching microsecond to millisecond timescales for large, complex systems [3, 4]. This success has generated a corresponding challenge: The immense volume and complexity of the data produced create a formidable bottleneck in analysis and interpretation.

The extraction of meaningful biophysical insight requires integrating a suite of well‐established computational analyses. These include fundamental metrics such as root‐mean‐square deviation (RMSD) to assess structural stability, root‐mean‐square fluctuation (RMSF) to profile flexibility, and radius of gyration (Rg) to measure global compactness [5]. Further characterization often involves hydrogen bond analysis, secondary structure assignment, solvent‐accessible surface area (SASA) calculations, and distilling high‐dimensional conformational space using clustering and dimensionality reduction techniques like principal component analysis (PCA) and t‐distributed stochastic neighbor embedding (t‐SNE) [6, 7]. More specialized analyses, such as the fraction of native contacts (Q‐value) for protein folding studies [8] and dihedral angle analysis with Ramachandran plots are also essential for comprehensive biophysical characterization.

While powerful low‐level libraries exist to perform these tasks, they operate in isolation. Tools such as MDTraj [9] and MDAnalysis [10] provide comprehensive Python APIs for trajectory manipulation, but require users to write and maintain complex, lengthy scripts to chain analyses and generate publication‐quality output. This fragmented landscape forces researchers to become script integrators, leading to a steep learning curve, significant time investment, and, most critically, substantial barriers to reproducibility. Inconsistent atom selections, alignment procedures, or algorithm parameters between bespoke scripts can yield different results, undermining direct comparison between studies and the scientific method itself [11]. General‐purpose workflow engines such as Snakemake [12] and Nextflow [13] provide powerful tools for pipeline automation and can help organize MD analyses. However, they do not embed MD‐specific reproducibility guarantees, such as consistent atom selections across all analyses, automatic alignment reuse, and deterministic random seeding for stochastic algorithms, as intrinsic design features. These must be manually implemented and verified by the user, reintroducing the very scripting burden FastMDAnalysis seeks to eliminate.

The need, therefore, is not merely for another analysis tool but for a cohesive computational framework that transforms this fragmented process into a standardized, reproducible, and automated workflow. Such a framework must prioritize reproducibility and usability without sacrificing the performance and accuracy of its underlying computational engines. It should enable both novice and expert researchers to execute complex, multi‐method studies that are currently impeded by scripting overhead.

Here, we present FastMDAnalysis (Fully Automated System for Molecular Dynamics Analysis), a novel software framework that establishes an integrated methodology for reproducible MD trajectory analysis. FastMDAnalysis is an open‐source Python package that bridges the gap between low‐level libraries and rigorous, end‐to‐end analysis. It serves as a high‐level orchestrator, unifying essential analyses through a coherent, object‐oriented interface mirrored by a command‐line interface. Its core contributions represent an advance in computational practice:

(i) A reproducibility‐by‐design architecture that enforces parameter consistency, provides comprehensive execution logging, and generates standardized, machine‐readable outputs for full provenance tracking, with an operational definition of reproducibility enforced through configuration locking, deterministic random seeding, and automatic dependency versioning; (ii) Comprehensive workflow automation that integrates trajectory loading, a modular and extensible suite of core analysis modules (RMSD, RMSF, Rg, hydrogen bonds, SASA, secondary structure, clustering, dimensionality reduction, fraction of native contacts, and dihedral angle analysis), and publication‐quality figure generation into a single, deterministic process; (iii) A dual‐interface design offering both an intuitive command‐line interface for batch processing and a Python API for interactive analysis, with consistent behavior across interfaces including flexible selective execution via include/exclude options and native support for all major trajectory formats (GROMACS [14], AMBER [15], CHARMM [16], etc.); and (iv) A harmonized API and structured output that produces directly comparable results across different analytical methods, facilitating benchmarking and multi‐method studies (see Section 2.4 for a detailed comparison with existing tools).

In this manuscript, we describe the architecture of FastMDAnalysis, validate its numerical accuracy and performance against established benchmarks, and demonstrate its utility through a case study. By providing a rigorous, integrated alternative to ad hoc scripting, FastMDAnalysis advances the methodology of computational analysis, making sophisticated, reproducible MD studies more accessible and efficient.

## 2 Software Description

### 2.1 Architecture and Design Overview

FastMDAnalysis employs a modular, object‐oriented architecture designed around four core principles: Reproducibility, usability, performance, and extensibility. The software is structured as a standard Python package with clean separation of concerns across logical components: A core analysis library (src/fastmdanalysis/), a comprehensive test suite (tests/), example workflows (examples/), and bundled datasets (src/fastmdanalysis/data/).

The central orchestrator is the FastMDAnalysis class, which implements a unified interface for workflow management and analysis coordination. Upon instantiation, the class loads trajectory data (supporting single files, file lists, or glob patterns) along with corresponding topology information. A key design innovation is the consistent application of global defaults for frame selection (start, stop, stride) and atom selection using MDTraj's powerful selection language (e.g., “protein and name CA”) [9]. These defaults persist throughout the instance's lifetime, ensuring that all analyses operate on identical data subsets—a foundational feature for reproducible, comparable results.

Supported trajectory formats include .xtc, .trr, .dcd, .nc, .h5, .lh5, .binpos, .xyz, and all other formats supported by MDTraj. Supported topology formats include .gro, .top, .pdb, .psf, .mol2, .h5, .lh5, and others. File format is automatically detected from the file extension, and no special configuration is required for GROMACS, AMBER, CHARMM, or other common simulation package outputs.

FastMDAnalysis strategically leverages established, high‐performance open‐source libraries to ensure computational robustness while focusing its innovation on workflow orchestration: MDTraj for trajectory I/O and fundamental geometric calculations [9]; scikit‐learn [17] and SciPy [18] for clustering algorithms (DBSCAN [19], K‐means, hierarchical clustering) and dimensionality reduction techniques (PCA, MDS, t‐SNE [20]); Matplotlib for publication‐quality figures [21]; and NumPy/SciPy for numerical computations [18, 22]. By building upon these trusted libraries, FastMDAnalysis delivers a reproducible and integrated methodological layer without compromising numerical accuracy or performance.

### 2.2 Core Analysis Modules

Each analytical routine is implemented as a self‐contained class within the analysis subpackage, each inheriting from a common BaseAnalysis abstract base class. This design ensures uniformity and extensibility, allowing advanced users and contributors to add new analysis modules with minimal boilerplate by subclassing BaseAnalysis and implementing a standard interface. The software currently provides ten core analysis modules: *RMSD Analysis*. The rmsd() method computes root‐mean‐square deviation relative to a reference structure after optimal superposition; *RMSF Analysis*. The rmsf() method calculates root‐mean‐square fluctuation of each atom about its time‐averaged position; *RG Analysis*. The rg() method computes the radius of gyration as a measure of global compactness; *Hydrogen Bond Analysis*. The hbonds() method identifies hydrogen bonds using the Baker–Hubbard geometric criteria [23], with automatic bond reconstruction for incomplete topologies; *Secondary Structure Analysis*. The ss() method assigns secondary structure elements via the DSSP algorithm [24]; *Solvent Accessible Surface Area Analysis*. The sasa() method computes SASA using the Shrake–Rupley algorithm [25]; *Clustering Analysis*. The cluster() method partitions conformational ensembles using DBSCAN, K‐means, or hierarchical clustering; *Dimensionality Reduction Analysis*. The dimred() method projects high‐dimensional data using PCA, MDS, or t‐SNE for visualization; *Fraction of Native Contacts Analysis*. The qvalue() method computes the fraction of native contacts (Q‐value), a collective coordinate widely used in protein folding studies to quantify structural similarity to a reference native state [8]; and *Dihedral Angle Analysis*. The dihedrals() method calculates backbone dihedral angles (ϕ, ψ, ω) across the trajectory. The module generates Ramachandran plots (ϕ–ψ distributions).

In addition to these core analysis modules, FastMDAnalysis is designed for continuous extension. The BaseAnalysis abstract base class provides a well‐defined interface for community‐contributed modules. Planned modules for future releases include contact map analysis, free energy landscape construction, principal component analysis of dihedral space (dPCA), and ensemble comparison metrics. This extensibility ensures that FastMDAnalysis can evolve alongside the methodological needs of the MD community.

### 2.3 Workflow Orchestration and Integration

The analyze() method embodies the core integrative innovation of FastMDAnalysis. It orchestrates the entire workflow through optimized coordination: Resolving input files, enforcing consistent data selection, reusing alignments to avoid redundant computation, and constructing shared feature matrices for downstream analysis. Each module executes with validated parameters, producing standardized data tables, publication‐quality figures, and comprehensive logs. This orchestration transforms a series of disjointed steps into a single, reproducible process, ensuring analytical coherence and full provenance tracking.

To provide users with greater control over visual output, FastMDAnalysis includes configurable plotting options. Time‐series plots (e.g., RMSD, RG, total SASA) display the raw trajectory trace by default. Statistical summaries, such as the global mean and standard deviation, can be optionally selected and included in the generated figures and data tables. Future releases will extend these capabilities to ensemble‐based statistical summaries.

### 2.4 Comparison With Existing Tools

FastMDAnalysis occupies a distinct niche in the molecular dynamics analysis ecosystem. While several excellent tools exist for trajectory analysis and workflow construction, none simultaneously combine (i) high‐level, end‐to‐end workflow orchestration, (ii) reproducibility‐by‐design enforced at the framework level, and (iii) a dual Python/CLI interface with consistent behavior. Table 1 summarizes the conceptual and functional distinctions between FastMDAnalysis and related software.

**TABLE 1:** Conceptual and functional comparison of FastMDAnalysis with existing MD analysis and workflow tools.

Tool	Primary role	Orchestration	Reproducibility by design	Dual interface	Scope
MDTraj [9]	Library	No	No	No	Trajectory I/O, MD analysis
MDAnalysis [10]	Library	No	No	No	Trajectory I/O, MD analysis
PyEMMA [26]	Library	No	No	No	Markov state modeling
MSMBuilder [27]	Library	No	No	No	Markov state modeling
PLUMED [7]	Library/Driver	Partial	No	No	Enhanced sampling, collective variables
Snakemake [12]	Workflow engine	Yes	Partial	No	General workflow automation
Nextflow [13]	Workflow engine	Yes	Partial	No	General workflow automation
FastMDAnalysis	Orchestrator	Yes	Yes	Yes	Unified automated MD analysis

#### 2.4.1 Orchestration Vs. Library Functionality

Tools such as MDTraj [9] and MDAnalysis [10] are foundational, low‐level libraries that provide comprehensive APIs for trajectory manipulation and geometric calculations. However, they are not workflows; they require users to write and maintain custom scripts to chain analyses, manage state, and generate outputs. FastMDAnalysis builds upon these libraries as computational backends, but contributes a high‐level orchestration layer that automates the entire analytical pipeline, from trajectory loading to figure generation, while enforcing parameter consistency and execution logging.

#### 2.4.2 Reproducibility‐by‐Design

General‐purpose workflow engines such as Snakemake [12] and Nextflow [13] excel at pipeline automation and can be used to construct MD workflows. They provide excellent tools for dependency management, containerization, and execution logging. However, MD‐specific reproducibility guarantees—such as consistent atom selections across all analyses, automatic alignment reuse, and deterministic random seeding for stochastic algorithms—are not intrinsic to their design; these must be manually implemented and verified by the user. FastMDAnalysis embeds these MD‐specific guarantees directly into its architecture.

#### 2.4.3 Specialized Analysis Frameworks

PyEMMA [26] and MSMBuilder [27] are powerful libraries for constructing Markov state models and analyzing conformational kinetics. They are specialized for a specific class of analyses and do not aim to provide general‐purpose trajectory analysis workflows. PLUMED [7] offers driver‐level automation for enhanced sampling and collective variable calculations, but its primary focus is on biasing simulations rather than post‐simulation trajectory analysis.

#### 2.4.4 Dual‐Interface Design

A distinguishing feature of FastMDAnalysis is its dual, consistent interface. Some tools offer a Python API (e.g., MDTraj, MDAnalysis, PyEMMA), while others provide a command‐line interface (e.g., cpptraj [5], PLUMED). However, maintaining behavioral parity between the two requires significant effort, and few tools offer both with identical semantics. FastMDAnalysis was designed with a unified internal dispatcher, ensuring that every operation accessible via the Python API is also available via the CLI with identical behavior. This lowers the barrier for both interactive exploration and high‐throughput batch processing, and is particularly valuable for teaching and for researchers transitioning from scripting to automated workflows.

### 2.5 Command‐Line and Python Interfaces

A cornerstone of FastMDAnalysis is its dual‐interface design, which provides consistent access to all functionalities both programmatically and via the command line. The Python API supports interactive exploration and integration into larger pipelines. For example, performing a full analysis on the bundled Trp‐cage trajectory [28] requires only:

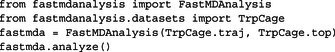

The unified command line interface (CLI), accessed via fastmda analyze, enables batch processing and high‐throughput workflows. The equivalent single CLI command to perform all available analyses on the Trp‐cage trajectory is:

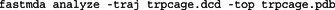

The ‐traj and ‐top arguments accept any trajectory or topology format natively supported by MDTraj, including GROMACS formats (.xtc, .trr, .gro, .top), AMBER formats (.nc, .prmtop, .inpcrd, .rst7), and CHARMM formats (.dcd, .psf, .crd, .pdb). For example:

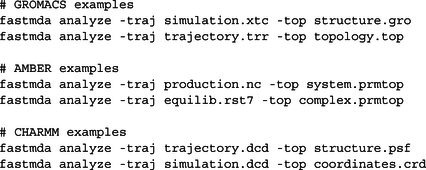

By default, analyze() executes all core analysis modules. For workflows requiring only a subset of analyses, FastMDAnalysis provides flexible selective execution through include and excludeoptions, which behave identically in both the Python API and the command‐line interface. In the Python API, the analyze() method accepts optional include and exclude parameters, each taking a list of analysis names:

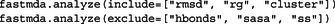

The command‐line interface provides directly corresponding –include and –exclude flags, accepting comma‐separated lists:

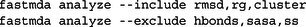

This design gives users full control over workflow scope while maintaining the reproducibility benefits of the unified orchestration layer.

### 2.6 Reproducibility Framework

In FastMDAnalysis, we adopt a rigorous, operational definition of reproducibility: Given identical input data and configuration parameters, the software must produce numerically equivalent outputs across different runs, platforms, and execution environments. This level of determinism is essential for auditable, trustworthy computational research and is enforced throughout the framework by multiple complementary mechanisms. *User‐customizable configuration* of YAML files specifying desired settings for any analysis module (e.g., clustering method, RMSD reference frame, plot styling). When supplied via the –options flag, these user‐provided values override the corresponding defaults. The software then merges the built‐in defaults with the user overrides in memory to produce the final configuration used for analysis. *Comprehensive execution logging* is automatically generated for every invocation. Each timestamped log file records: (i) the full command or API call; (ii) the versions of FastMDAnalysis and all core dependencies (MDTraj, scikit‐learn, NumPy, SciPy, Matplotlib); (iii) the precise start and stop times of each analysis module; and (iv) any warnings or errors encountered. These logs serve as a complete provenance trace, capturing the exact workflow and user specifications, enabling independent verification and facilitating debugging. *Deterministic random number management* is enforced for all stochastic algorithms. FastMDAnalysis explicitly sets and propagates fixed random seeds for t‐SNE, K‐means++ initialization, and other nondeterministic procedures. This guarantees that repeated runs yield identical embeddings and cluster assignments, as validated in Section 3.1 and Table 2. *Standardized, machine‐readable output formats* are used for all numerical results. For each auto‐generated figure, FastMDAnalysis writes a corresponding .dat file containing the underlying tabular data (e.g., RMSD time series, cluster assignments, per‐residue SASA values). These plain text files are structured with clear headers and column definitions, enabling users to re‐plot, re‐analyze, or verify results without re‐executing the entire workflow. Combined with the execution logs, this provides full downstream provenance tracking and facilitates independent validation. These design choices ensure that any analysis can be exactly rerun, audited, or validated, directly addressing a critical need in MD trajectory analysis.

**TABLE 2:** Comprehensive numerical validation of FastMDAnalysis against reference implementations^a^.

Analysis module	Backend	RMSE	Status	Tolerance margin	Validation details
RMSD	MDTraj	0.0	Excellent	$>10^{4}\times$	Perfect numerical agreement across 500 frames
RMSF	MDTraj	0.0	Excellent	$>10^{4}\times$	Identical per‐atom fluctuations (304 atoms)
Radius of Gyration	MDTraj	$2.71\times 10^{-9}$	Excellent	36,900×	Near machine precision (target: $1\times 10^{-4}$)
Hydrogen Bonds	MDTraj	0.0	Excellent	$>10^{4}\times$	Identical hydrogen bond identification
Secondary Structure	MDTraj	—	Excellent	Exact match	100% categorical agreement (10,000 assignments)
SASA (total)	MDTraj	0.0	Excellent	$>10^{4}\times$	Perfect agreement in total solvent accessibility
SASA (per‐residue)	MDTraj	0.0	Excellent	$>10^{4}\times$	Identical residue‐level solvent accessibility
SASA (average)	MDTraj	0.0	Excellent	$>10^{4}\times$	Consistent time‐averaged residue SASA
PCA	scikit‐learn	0.0	Excellent	$>10^{4}\times$	Identical principal components and projections
MDS	scikit‐learn	0.0	Excellent	$>10^{4}\times$	Consistent multidimensional scaling
t‐SNE	scikit‐learn	0.0	Excellent	$>10^{4}\times$	Identical nonlinear embeddings
K‐means	scikit‐learn	0.0	Excellent	$>10^{4}\times$	Identical cluster assignments (500 frames)
DBSCAN	scikit‐learn	0.0	Excellent	$>10^{4}\times$	Consistent density‐based partitioning
Hierarchical	SciPy	0.0	Excellent	$>10^{4}\times$	Identical dendrogram‐based assignments

^a^ Validation criteria: *Excellent Agreement* = RMSE $<1\times 10^{-4}$, *Good Agreement* = RMSE $<1\times 10^{-2}$. All analyses achieved “Excellent” status with significant margins, demonstrating numerical equivalence to reference implementations. System: Trp‐cage miniprotein (PDB ID: 1L2Y), 500 frames subsampled from a 100 ns simulation comprising 304 protein atoms across 20 residues. Comparisons against MDTraj (v1.11.0), scikit‐learn (v1.7.2), and SciPy (v1.13.1). Tolerance margin is defined as the ratio ${\text{RMSE}}_{\text{tol}}/{\text{RMSE}}_{\text{observed}}$, with ${\text{RMSE}}_{\text{tol}}=10^{-4}$ for “Excellent” agreement. For quantities with RMSE below the printed precision (0.0), we report a conservative lower bound (e.g., “$>10^{4}\times$”) rather than an infinite margin.

## 3 Validation and Benchmarks

### 3.1 Accuracy Validation

To establish the scientific validity and reliability of FastMDAnalysis as a computational framework, we conducted comprehensive numerical benchmarking against direct reference implementations using identical computational backends. This validation demonstrates that our orchestration layer introduces no numerical distortion while providing reproducibility and workflow integration. The validation employed the Trp‐cage miniprotein (PDB ID: 1L2Y) as a model system, analyzing 500 frames subsampled from a 100 ns simulation ($N_{\text{frames}}$ = 5000, stride = 10) comprising 304 protein atoms across 20 residues [28, 29].

Quantitative comparisons were performed using multiple metrics: Root mean square error (RMSE), maximum absolute difference, mean absolute difference, and statistical distribution analysis. All core analysis modules demonstrated excellent agreement with reference implementations, exceeding tolerance thresholds by orders of magnitude (Table 2). Structural metrics, including RMSD and RMSF, showed near‐perfect numerical identity (RMSE $<10^{-12}$), while radius of gyration calculations achieved near‐machine precision (RMSE = $2.71\times 10^{-9}$ nm). Secondary structure assignments via the DSSP algorithm achieved 100% categorical agreement across all frames and residues (10,000 elements total).

Solvent‐accessible surface area calculations exhibited perfect fidelity across all aggregation levels, with total SASA, per‐residue SASA, and averaged per‐residue SASA all showing differences below floating‐point precision (RMSE $<10^{-12}$). Hydrogen bond identification using the Baker–Hubbard algorithm detected identical bond networks (RMSE = 0.0), while clustering algorithms (K‐means, DBSCAN, Hierarchical) produced indistinguishable membership assignments. PCA and MDS achieved numerical agreement (RMSE $<10^{-12}$) due to their deterministic algorithms. t‐SNE embeddings were identical across implementations when using fixed random seeds, ensuring reproducibility as shown in Table 2.

The numerical agreement across all modules (Table 2) confirms that FastMDAnalysis serves as a faithful orchestrator, preserving the accuracy of underlying libraries while adding reproducibility and integration capabilities. This validation ensures researchers can adopt the framework without compromising scientific rigor. By demonstrating numerical equivalence with established libraries including MDTraj, scikit‐learn, and SciPy, FastMDAnalysis delivers seamless workflow integration while maintaining full computational accuracy. The comprehensive validation framework included in the Supporting Information enables independent reproducibility testing, ensuring consistent and reliable performance across diverse computational environments and use cases.

### 3.2 Performance and Usability Benchmark

While numerical accuracy is essential, a primary objective of FastMDAnalysis is to transform MD analysis from a scripting‐intensive process into an efficient, standardized methodology. To quantitatively assess this transformation, we benchmarked FastMDAnalysis against the direct scripting approach using MDTraj and MDAnalysis. The tests were performed on the native‐state ensemble of ubiquitin (a 5×50 ns simulation, PDB ID: 1UBQ) from a previous protein conformational ensemble study by Aina et al. [30], using the full 5000‐frame trajectory available on Zenodo [31]. For scaling, we considered subsets of the first 500, 1000, 2000, 3000, 4000, and 5000 frames from this dataset.

#### 3.2.1 Benchmark Design

All analyses were executed via the respective Python APIs with matched parameters, atom selections (protein), and frame ranges. For each library and frame count, we ran a standard analysis pipeline consisting of (i) trajectory loading, frame and atom selection, (ii) RMSD, RMSF, and Rg calculations, (iii) K‐means clustering with k=3 on Cartesian coordinates, and (iv) generation of the corresponding plots and data files. Computational performance was quantified as wall–clock runtime and peak resident memory (RSS), averaged over five independent replicates. Code complexity was quantified as the number of effective lines of code (LOC), defined as non‐blank, non‐comment statements in the workflow functions and required helpers used to reproduce the same set of figures and output files for each library.

#### 3.2.2 Code Complexity

**FIGURE 1:**
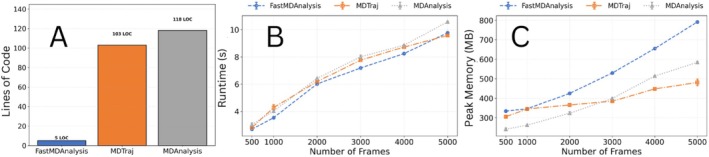
Performance and usability benchmarks for molecular dynamics analysis libraries on the ubiquitin dataset. (A) Effective lines of code (LOC) required to implement the full analysis pipeline (RMSD, RMSF, Rg, and K‐means clustering, including figure generation) using the Python APIs. FastMDAnalysis requires only 5 LOC to express the workflow, compared to 103 LOC for MDTraj and 118 LOC for MDAnalysis. (B) Scaling of mean wall–clock time with the number of frames (n=5 replicates per point). All three libraries exhibit approximately linear scaling in trajectory length. FastMDAnalysis tracks closely with MDTraj across all frame counts and remains within the same performance envelope as MDAnalysis. (C) Scaling of peak memory usage (resident set size) with the number of frames. Memory usage grows approximately linearly for all libraries. FastMDAnalysis incurs a moderately higher peak footprint than MDTraj and MDAnalysis, reflecting its array‐resident orchestration and integrated plotting, but all three remain in a comparable range even at 5000 frames. Error bars represent the standard error of the mean.

Figure 1A highlights the substantial reduction in user‐facing code volume achieved by FastMDAnalysis. Implementing the full workflow with MDTraj and MDAnalysis required 103 and 118 effective lines of code, respectively, including the explicit plotting and clustering logic needed to reproduce the same figures and data artifacts. The corresponding FastMDAnalysis workflow can be expressed in only 5 LOC via the high‐level Python API. This represents a reduction in code volume of ∼95% relative to MDTraj and ∼96% relative to MDAnalysis. The same analysis can also be executed, using FastMDAnalysis CLI, with a single self‐documenting command. This drastic simplification reduces boilerplate, lowers the risk of implementation errors, and improves reproducibility for students and non‐specialists.

#### 3.2.3 Computational Performance

Despite its higher level of abstraction, FastMDAnalysis maintains performance parity with direct MDTraj and MDAnalysis scripts. As shown in Figure 1B, runtime scales approximately linearly with the number of frames for all three libraries. Across the full range from 500 to 5000 frames, FastMDAnalysis closely tracks MDTraj, with differences that are small relative to the overall runtime and typically within the error bars of the five‐replicate averages. MDAnalysis is slightly slower at the largest frame counts but remains in the same order of magnitude. These results demonstrate that the convenience and usability advantages provided by FastMDAnalysis do not come at the cost of computational efficiency.

#### 3.2.4 Memory Footprint

Figure 1C summarizes the scaling of peak resident memory with trajectory length. All three libraries show approximately linear growth in peak memory usage as a function of frame count. FastMDAnalysisexhibits the highest peak memory at each frame count, reflecting its design choice to keep intermediate arrays and clustering features in memory while orchestrating analysis and plotting in a single environment. However, the differences are moderate: at 5000 frames, FastMDAnalysis reaches a higher peak memory than MDAnalysis, which in turn exceeds MDTraj, but all three toolkits remain within a practically similar range.

Together, these benchmarks demonstrate that FastMDAnalysis delivers methodological transformation, reducing code volume by >90% while maintaining performance parity. This combination makes complex, multi‐method analyses practically accessible, addressing a key bottleneck in MD analysis workflows. The framework enables researchers to focus on scientific interpretation rather than script development, without sacrificing numerical accuracy or computational efficiency.

## 4 Case Study

### 4.1 Rapid Analysis of Protein Conformational Dynamics

To demonstrate the practical impact and methodological advantage of FastMDAnalysis in realistic research scenarios, we present a comprehensive analysis of Bovine Pancreatic Trypsin Inhibitor (BPTI), a model system extensively characterized in both experimental and computational studies [32, 33]. This case study illustrates how FastMDAnalysis enables complex, multi‐method analysis that would be cumbersome with traditional scripting approaches. We analyzed a 100 ns molecular dynamics (MD) trajectory of BPTI (PDB ID: 6PTI) [34, 35] using FastMDAnalysis's standardized workflow, requiring minimal coding expertise.

#### 4.1.1 System Preparation and Simulation

The BPTI structure (PDB ID: 6PTI) was curated to remove non‐water heteroatoms, with protonation states assigned at physiological pH (7.0). The native disulfide bond network (Cys5–Cys55, Cys14–Cys38, and Cys30–Cys51) was preserved from the crystal structure. The system was parameterized with the CHARMM36m [36] protein force field and the TIP3P water model. Missing atoms and hydrogens were added using PDBFixer. Simulations were performed using OpenMM 8.2 [37] under periodic boundary conditions in a cubic box with 1.0 nm solvent padding and 0.15 M NaCl. Long‐range electrostatics were treated with particle‐mesh Ewald (PME) using a real‐space cutoff of 1.0 nm, and Lennard‐Jones interactions employed a force‐based switching function between 1.0 and 1.2 nm. Hydrogen bonds were constrained using the LINCS algorithm [38] with hydrogen mass repartitioning (1.5 amu), enabling a 2 fs timestep. Temperature was maintained at 300 K using a Langevin middle integrator (friction coefficient 1.0 ps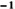), and pressure was controlled at 1 bar using a Monte Carlo barostat (attempted exchanges every 50 steps). After energy minimization (5000 steps), the system underwent NVT equilibration for 500 ps followed by NPT equilibration for 2.0 ns before a 100 ns production run, with frames saved every 10 ps for a total of 10,000 frames.

#### 4.1.2 Automated Analysis With FastMDAnalysis

The entire analysis pipeline, including eight distinct analytical methods, was executed through a single, reproducible command:

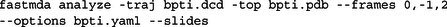

The options.yaml configuration file defined parameters for hierarchical clustering (k=6) and PCA as the method for dimensionality reduction. This integrated workflow completed in under 5 min on a standard laptop (2021 MacBook Pro, M1 Pro, 32 GB RAM), demonstrating both computational efficiency and the framework's ability to coordinate complex, multi‐method study seamlessly.

#### 4.1.3 Structural Stability and Fluctuation

**FIGURE 2:**
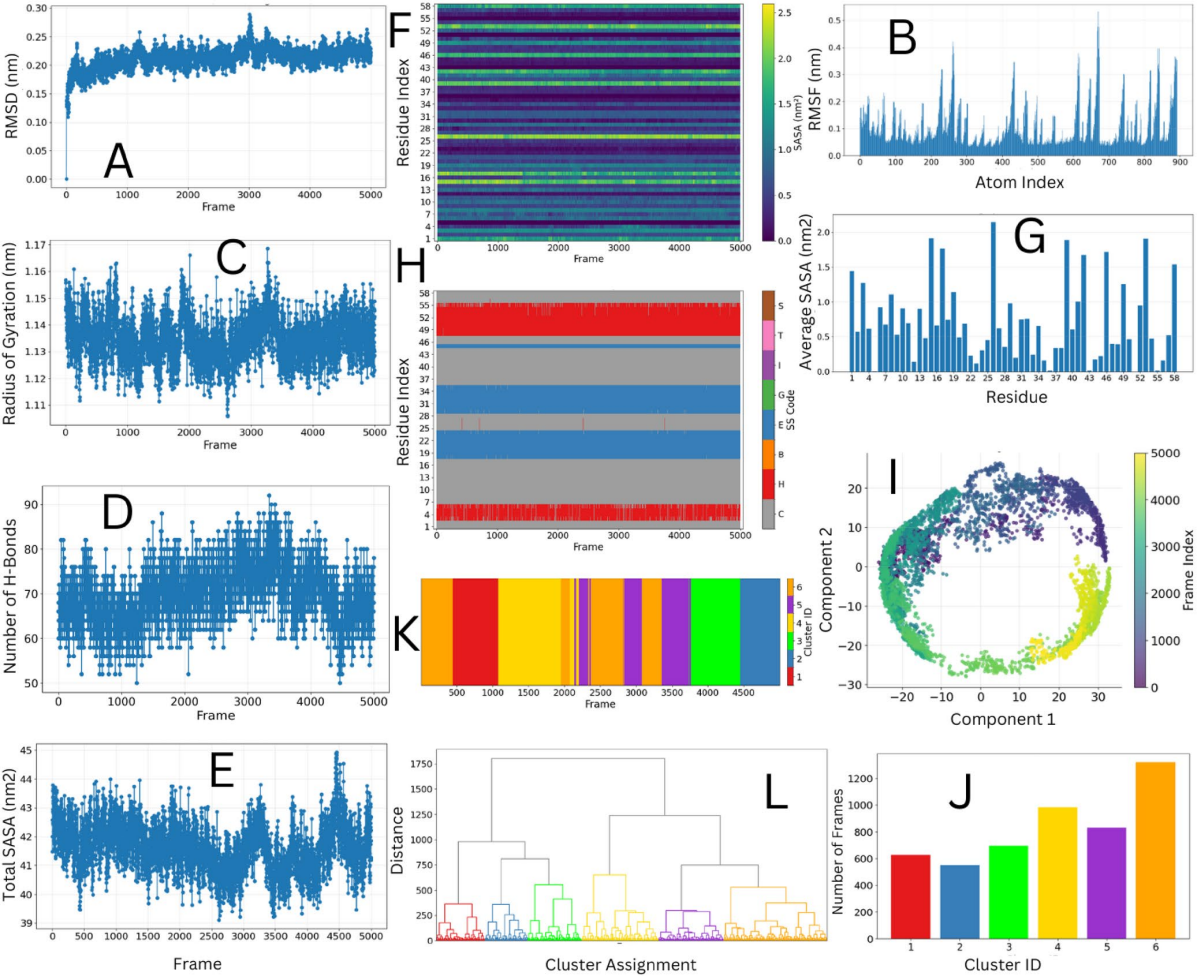
Comprehensive conformational analysis of Bovine Pancreatic Trypsin Inhibitor (BPTI) from a 100 ns molecular dynamics simulation analyzed using FastMDAnalysis. (A) Backbone RMSD relative to the initial structure (reference frame 0, aligned) shows rapid equilibration and stable dynamics with an average of 0.22±0.03 nm. (B) Atomic flexibility quantified by RMSF. The profile shows a stable, rigid core (low RMSF) punctuated by specific high‐flexibility loops. (C) Radius of gyration maintains constant compactness at 1.14±0.02 nm throughout the simulation, reflecting structural constraints from three disulfide bonds. (D) Hydrogen bond count remains stable at 72±6 per frame, indicating preserved secondary structure integrity. (E) Total solvent accessible surface area (SASA) shows minimal fluctuation around 41.8 nm^2^ throughout the trajectory. (F) Per‐residue SASA vs. frame reveals dynamic solvent exposure patterns, with loop regions showing higher variability. (G) Average per‐residue SASA profile identifies consistently exposed and buried residues. (H) Secondary structure timeline (DSSP) using standard codes: H (α‐helix), E (extended strand/β‐sheet), G (3–10 helix), I (π‐helix), T (turn), S (bend), and C (coil/loop). Analysis reveals persistent β‐sheet content (residues 18–24, 29–35) and stable C‐terminal helix (residues 48–55). (I) Principal component analysis captures a ring‐like manifold of conformational variance. (J) Hierarchical cluster populations (6 clusters) demonstrate conformational states with populations of 26%, 20%, 17%, 14%, 13%, and 11%. (K) Hierarchical clustering trajectory histogram shows the temporal distribution of conformational states across the simulation. (L) Hierarchical clustering dendrogram illustrates the distance relationships and frame assignments between conformational states. All analyses were executed via a single FastMDAnalysis command, demonstrating rapid, reproducible, and comprehensive MD trajectory analysis.

The RMSD analysis relative to the initial frame revealed rapid equilibration within the first 5 ns, maintaining stability with an average backbone RMSD of 0.22±0.03 nm throughout the production phase (Figure 2A), indicating structural integrity consistent with BPTI's known rigidity. RMSF analysis reveals the expected dynamic profile for a compact, disulfide‐stabilized protein (Figure 2B). Most of the structure exhibits high rigidity (RMSF <0.1 nm), forming a stable baseline that indicates a well‐structured core, punctuated by sharp peaks (RMSF ≈0.3–0.5 nm) corresponding to loop regions and termini.

#### 4.1.4 Global Compactness and Solvent Accessibility

The radius of gyration remained constant at 1.14±0.02 nm throughout the simulation (Figure 2C), reflecting the structural constraints imposed by BPTI's three disulfide bonds. Total SASA values were stable at approximately 41.8±1.0 nm^2^(Figure 2E), with per‐residue SASA profiles highlighting solvent‐exposed loop regions and shielded core residues (Figure 2G). The per‐residue SASA vs. frame analysis reveals dynamic solvent exposure patterns throughout the trajectory (Figure 2F), with loop regions showing higher variability while core residues maintain consistent burial.

#### 4.1.5 Hydrogen Bonding and Secondary Structure

The number of hydrogen bonds per frame remained stable at 72±6 (Figure 2D), consistent with maintained secondary structure. DSSP‐based secondary structure analysis revealed remarkable stability in BPTI's structural elements, with β‐sheet content (residues 18–24, 29–35) and the C‐terminal helix (residues 48–55) preserved throughout the simulation (Figure 2H).

#### 4.1.6 Dimensionality Reduction and Conformational Clustering

Principal Component Analysis reveals a temporally ordered ring‐like manifold in the subspace of the first two principal components (Figure 2I), indicating a continuous, cyclic conformational transition rather than simple stochastic fluctuation. Hierarchical clustering identified six metastable conformational states with heterogeneous populations (26%, 20%, 17%, 14%, 13%, and 11%) (Figure 2J), with the dendrogram illustrating the distance relationships and frame assignments between these conformational states (Figure 2L). The temporal evolution shows long‐lived, contiguous assignments separated by infrequent transitions (Figure 2K), confirming metastable character. This skewed distribution indicates limited conformational diversity, consistent with BPTI's disulfide‐constrained rigidity.

This case study demonstrates that FastMDAnalysis transforms MD analysis from a fragmented scripting task into an integrated, efficient methodology. The framework enabled a comprehensive investigation of BPTI's dynamics, validating its known stability while revealing subtle conformational transitions through a single, reproducible command. By making complex, multi‐method analyses both accessible and reproducible, FastMDAnalysis addresses critical needs for reproducibility and efficiency in computational structural biology.

## 5 Conclusion

We have introduced FastMDAnalysis, a unified framework that establishes a reproducible, automated methodology for molecular dynamics trajectory analysis. By integrating a comprehensive and extensible suite of core analysis modules into a single, coherent environment, it transforms a traditionally fragmented, script‐dependent process into an efficient and standardized workflow. The framework's rigorous adherence to reproducibility‐by‐design principles, through consistent parameter management, detailed execution logging, and standardized outputs, ensures that analyses are transparent, repeatable, and trustworthy.

Built upon the robust, high‐performance foundations of MDTraj, scikit‐learn, and SciPy, FastMDAnalysis delivers numerical accuracy alongside transformative usability. Our validation confirms numerical equivalence to reference implementations, while performance benchmarks demonstrate a >90% reduction in code volume without compromising computational efficiency. The software natively supports all major trajectory and topology formats, including GROMACS, AMBER, CHARMM, and others, via MDTraj's backend, with automatic format detection. The case study illustrates how the framework enables complex, multi‐method analysis through a single command, extracting comprehensive biophysical insights in minutes rather than hours and days of script development.

By eliminating scripting barriers and embedding reproducibility into the analytical workflow, FastMDAnalysis provides a methodological advance that makes rigorous, integrated MD studies accessible to both novice and expert researchers. Its modular architecture, built around the BaseAnalysis abstract base class, invites community extension and positions the framework to grow alongside the methodological needs of the field. The current framework focuses on orchestrating established analyses for single trajectories, with its scope defined by the capabilities of the underlying libraries. Future development will aim to include more specialized methods (e.g., contact maps, free energy landscapes, dihedral PCA) and support for comparative ensemble analysis with statistical summaries across replicates. The software is positioned to become a valuable resource for the computational chemistry, biology, and biophysics communities, enhancing productivity and fostering reproducible research practices. Its open‐source nature and modular architecture invite community participation and extension, paving the way for more accessible, reproducible, and efficient molecular dynamics analysis.

## Software Availability

*Repository*. https://github.com/aai‐research‐lab/fastmdanalysis.

*Archive*. The source code is archived at Zenodo (DOI: https://doi.org/10.5281/zenodo.17510591).

*Operating Systems*. Platform‐independent (Linux, macOS, Windows).

*Programming Language*. Python.

*Dependencies*. MDTraj, scikit‐learn, NumPy, SciPy, Matplotlib.

*License*. The software is available under the MIT license.

*Documentation*: Available at https://fastmdanalysis.readthedocs.io.

*Support*. Bug reports and feature requests are managed through the GitHub issue tracker.

## Author Contributions

**Adekunle Aina:** conceptualization; methodology; software; simulation; data analysis; writing – original draft; writing – review and editing; supervision; funding acquisition. **Derrick Kwan:** software; data analysis; visualization.

## Funding

This research was supported by startup funds from California State University Dominguez Hills (to A.A.).

## Conflicts of Interest

The authors declare no conflicts of interest.

## Supporting information




**Data S1: Validation details.** https://github.com/aai‐research‐lab/FastMDAnalysis/blob/validation/README.md. **Benchmark details**. https://github.com/aai‐research‐lab/FastMDAnalysis/blob/benchmark/README.md.

## Data Availability

The data that support the findings of this study are openly available in Zenodo at https://zenodo.org/records/17755442, reference number https://doi.org/10.5281/zenodo.17755442. All simulation datasets used in this study are available via Zenodo: Trp‐cage validation data (DOI: https://doi.org/10.5281/zenodo.17755214), BPTI case study data (DOI: https://doi.org/10.5281/zenodo.17568238), and ubiquitin benchmark data (DOI: https://doi.org/10.5281/zenodo.7792287).
