# Supplementary material for: FastMDAnalysis: Software for Automated Analysis of Molecular Dynamics Trajectories
Source: J Comput Chem. 2026 Mar 29;47(8):e70350. doi: 10.1002/jcc.70350 (PMC13033318; doi:10.1002/jcc.70350)
Supplement: Supplementary file 1 — Data S1: Validation details. https://github.com/aai‐research‐lab/FastMDAnalysis/blob/validation/README.md. Benchmark details. https://github.com/aai‐research‐lab/FastMDAnalysis/blob/benchmark/README.md. [file JCC-47-0-s001.pdf]

Supporting Information

FastMDAnalysis: Software for Automated Analysis of Molecular Dynamics Trajectories

Adekunle Aina[1,2,3] and Derrick Kwan[1,3]

[1]Department of Physics, California State University Dominguez Hills, CA 90747, USA
[2]Biophysics Program, California State University Dominguez Hills, CA 90747, USA
[3]AAI Research Lab, California State University Dominguez Hills, CA 90747, USA

Corresponding author: Adekunle Aina (aaina@csudh.edu)

Contents

1 Accuracy Validation Suite **2**
1.1 Step 1: Obtain the Validation Code . . . 2
1.2 Step 2: Install Dependencies . . . 2
1.3 Step 3: Run the Validation Script . . . 3
1.4 Validation Output Files . . . 3
1.4.1 JSON Report (`validation_report.json`) . . . 3
1.4.2 CSV Summary (`validation_summary.csv`) . . . 4
1.5 Analyses Performed . . . 4
1.5.1 Structural Metrics (MDTraj backends) . . . 4
1.5.2 Statistical Learning (scikit-learn and SciPy backends) . . . 5
1.6 Comparison Metrics and Acceptance Criteria . . . 5
1.7 Reproducibility Notes . . . 5

2 Performance Benchmark Suite **6**
2.1 Step 1: Obtain the Benchmark Code . . . 6
2.2 Step 2: Install Dependencies . . . 6
2.3 Step 3: Download and Prepare Data . . . 6
2.4 Step 4: Run Benchmarks . . . 6
2.5 Benchmark Output Files . . . 7
2.6 Benchmark Design Details . . . 7
2.6.1 Dataset . . . 7
2.6.2 Performance Scaling . . . 7
2.6.3 Workflow Complexity (Lines of Code) . . . 7
2.6.4 Verification . . . 8

References **9**

S1. Accuracy Validation Suite

This section describes the procedures for reproducing the numerical accuracy validation results reported in the manuscript (Section 3.1 and Table 2). The validation suite quantifies the numerical agreement between `FastMDAnalysis` (as published on PyPI) and established reference libraries (MDTraj [1], scikit-learn [3], SciPy [4]) on the Trp-cage miniprotein benchmark [10, 11].

S1.1. Step 1: Obtain the Validation Code

Create and activate a clean virtual environment, then check out the `validation` branch of the lab repository.

Using `conda` (recommended):

```
conda create -n fastmda_validation_env python=3.9
conda activate fastmda_validation_env
```

Clone the repository and switch to the validation branch:

```
git clone -b validation
   https://github.com/aai-research-lab/FastMDAnalysis.git
cd FastMDAnalysis
```

If the repository is already cloned locally:

```
git fetch origin
git checkout validation
```

S1.2. Step 2: Install Dependencies

Install the *published* `FastMDAnalysis` package from PyPI along with the minimal dependencies required by the validation script:

```
pip install --upgrade pip
pip install fastmdanalysis mdtraj numpy scipy scikit-learn
```

Important: Do *not* run `pip install -e .` in this environment. This ensures that `import fastmdanalysis` resolves to the **PyPI installation**, not the local source tree. MDAnalysis is *not* required for this validation workflow.

To closely match the published results, pin the exact library versions used in the JCC submission [1, 3, 4]:

```
pip install mdtraj==1.11.0 scikit-learn==1.7.2 scipy==1.13.1
```

S1.3. Step 3: Run the Validation Script

From the `FastMDAnalysis` repository root (on the `validation` branch):

```
python validate_fastmda.py
```

By default, this reproduces the JCC validation configuration:

- **System:** Trp-cage miniprotein (PDB ID: 1L2Y)
- **Trajectory:** bundled Trp-cage MD trajectory from the PyPI package
- **Frames:** `0:-1:10` (start = 0, end = last frame, stride = 10) → 500 frames subsampled from a 5,000-frame trajectory
- **Atom selection:** `protein` (304 protein atoms, 20 residues)
- **Output directory:** `validation_output/`

Optional parameters may be passed to change the frame range, atom selection, or output directory:

```
python validate_fastmda.py \
    --frames 0:-1:5 \
    --atoms "protein" \
    --output-dir validation_output_stride5
```

S1.4. Validation Output Files

After a successful run with default settings, the following files are generated in `validation_output/`:

- `validation_report.json` — Detailed, machine-readable report of all validation comparisons.
- `validation_summary.csv` — Human-readable summary (one row per comparison) with key statistics.

These files correspond directly to the numerical results summarized in Table 2 of the main manuscript.

JSON Report (`validation_report.json`)

Each entry in the JSON report includes the following fields (some fields vary by metric):

Field	Description
`name`	Analysis module (e.g., `RMSD`, `SASA (total)`)
`backend`	Reference implementation (`mdtraj`, `sklearn`, `scipy`)
`metric`	Metric identifier (e.g., `rmsd`, `total_sasa`)
`status`	Qualitative outcome (`pass`/`warn`/`fail`/ `error`/`info`)
`shape_match`	Boolean: result shapes are identical
`max_abs_diff`	Maximum absolute difference
`mean_abs_diff`	Mean absolute difference
`rmse`	Root mean square error
`mismatch_count`	Number of elements exceeding tolerance
`fastmda_stats`	Min, max, mean, std for FastMDAnalysis array
`ref_stats`	Min, max, mean, std for reference array
`fastmda_shape`	Array shape from FastMDAnalysis
`ref_shape`	Array shape from reference implementation
`detail`	Explanatory message (e.g., "Excellent agreement (RMSE=0.00e+00)")

CSV Summary (`validation_summary.csv`)

The CSV provides a compact view suitable for quick inspection, spreadsheets, and automated regression checks. It includes the following columns: `analysis_name`, `backend`, `metric`, `status`, `shape_match`, `max_abs_diff`, `mean_abs_diff`, `rmse`, `mismatch_count`, `fastmda_min`, `fastmda_max`, `fastmda_mean`, `fastmda_std`, `ref_min`, `ref_max`, `ref_mean`, `ref_std`, `fastmda_shape`, `ref_shape`.

Each row corresponds to a specific analysis/metric combination (e.g., RMSD vs. MDTraj, PCA vs. scikit-learn).

S1.5. Analyses Performed

The validation script tests all core analysis modules of `FastMDAnalysis` against direct calls to the underlying reference libraries using identical inputs and hyperparameters.

Structural Metrics (MDTraj backends)

- **RMSD** — Time series of backbone RMSD relative to a reference frame.
- **RMSF** — Per-atom root-mean-square fluctuation relative to the average structure.
- **Radius of gyration (R_g)** — Time series of mass-weighted R_g.
- **SASA** — Total SASA per frame, per-residue SASA per frame, and time-averaged per-residue SASA (Shrake–Rupley algorithm [7]).
- **Hydrogen bonds** — Per-frame hydrogen-bond counts using the Baker–Hubbard geometric criteria [5].
- **Secondary structure** — DSSP assignments [6] (simplified alphabet) per residue and frame.

Statistical Learning (scikit-learn and SciPy backends)

- **Dimensionality reduction:** PCA, Multidimensional Scaling (MDS), and *t*-distributed Stochastic Neighbor Embedding (t-SNE [9]); implemented via scikit-learn [3].
- **Clustering:** K-means, DBSCAN [8] (density-based), and hierarchical clustering (SciPy [4] `linkage` with Ward method and `fcluster`); K-means and DBSCAN via scikit-learn [3].

S1.6. Comparison Metrics and Acceptance Criteria

For each analysis, `FastMDAnalysis` results are compared to reference results using:

- Root mean square error (RMSE)
- Maximum absolute difference
- Mean absolute difference
- Number of elements exceeding a fixed tolerance
- Shape consistency checks

The script assigns qualitative labels based on RMSE and shape agreement:

Label	RMSE threshold	Interpretation
Excellent agreement	$< 1 \times 10^{-4}$	Near machine precision
Good agreement	$< 1 \times 10^{-2}$	Acceptable for most uses
`warn`	$\geq 1 \times 10^{-2}$	Larger discrepancy detected
`fail`	Shape mismatch or large error	Review required
`error`	Exception during computation	Review required

S1.7. Reproducibility Notes

- **System:** Trp-cage miniprotein (PDB ID: 1L2Y) [10, 11], 100 ns trajectory comprising 5,000 frames; validation uses 500 frames (stride = 10) and 304 protein atoms across 20 residues.
- **Reference library versions** used in the JCC submission: MDTraj 1.11.0 [1], scikit-learn 1.7.2 [3], SciPy 1.13.1 [4].

All core modules (RMSD, RMSF, R_g, SASA, hydrogen bonds, secondary structure, dimensionality reduction, and clustering) achieved **Excellent** agreement, with differences at or below numerical precision for most metrics (see Table 2 of the main manuscript).

S2. Performance Benchmark Suite

This section describes the procedures for reproducing the performance and workflow-complexity benchmarks reported in the manuscript (Section 3.2 and Figure 1). `FastMDAnalysis` is benchmarked against MDTraj [1] and MDAnalysis [2].

S2.1. Step 1: Obtain the Benchmark Code

Create and activate a dedicated virtual environment:

```
conda create -n fastmda_benchmark_env python=3.9
conda activate fastmda_benchmark_env
```

Clone the repository and switch to the benchmark branch:

```
git clone -b benchmark
   https://github.com/aai-research-lab/FastMDAnalysis.git
cd FastMDAnalysis
```

S2.2. Step 2: Install Dependencies

```
pip install fastmdanalysis mdtraj MDAnalysis numpy scipy \
            scikit-learn matplotlib psutil
```

S2.3. Step 3: Download and Prepare Data

The ubiquitin benchmark dataset is publicly available on Zenodo (DOI: 10.5281/zenodo.7792287).

1. Download the dataset from Zenodo.
2. Create a `Ubiquitin/` directory in the repository root.
3. Place the files with the following names:
   - Trajectory: `Ubiquitin/ubiquitin.dcd` (originally `Q99.dcd`)
   - Topology: `Ubiquitin/ubiquitin.pdb` (originally `topology.pdb`)

S2.4. Step 4: Run Benchmarks

Navigate to the data directory and execute the performance scaling benchmark:

```
cd Ubiquitin
python ../scripts/benchmark_scaling.py \
    --frames 500 1000 2000 3000 4000 5000 \
    --iterations 5 \
    --keep-artifacts 5000
```

Generate runtime and memory scaling plots:

```
python ../scripts/benchmark_plot.py
```

Analyze workflow code complexity:

```
python ../scripts/benchmark_loc.py
```

S2.5. Benchmark Output Files

After a successful run, the following files are generated in `benchmark_output/`:

File	Contents
`scaling_metrics.json`	Raw performance data (runtime and memory)
`combined_runtime.png`	Runtime scaling plot (Figure 1B in manuscript)
`combined_memory.png`	Memory scaling plot (Figure 1C in manuscript)
`benchmark_loc.png`	Lines of code comparison (Figure 1A in manuscript)
`*_artifacts/`	Analysis outputs for the 5,000-frame run

S2.6. Benchmark Design Details

Dataset

Benchmarks were performed on the native-state ensemble of ubiquitin (PDB ID: 1UBQ; a 5×50 ns simulation) from a previously published conformational ensemble study [12]. The full 5,000-frame trajectory is available on Zenodo (DOI: 10.5281/zenodo.7792287) [13]. Frame subsets of 500, 1,000, 2,000, 3,000, 4,000, and 5,000 frames were used to assess scaling behavior.

Performance Scaling

For each library and frame count, performance was measured over five independent replicates (`--iterations 5`). The standard analysis pipeline executed by all three libraries consisted of:

1. Trajectory loading, frame and atom selection (`protein`)
2. RMSD (aligned to first frame), RMSF, and R_g calculations
3. K-means clustering ($k = 3$) on Cartesian coordinates
4. Generation of corresponding plots and data files

Computational performance is reported as mean wall-clock runtime and peak resident memory (RSS), averaged over five replicates. Error bars in Figure 1 represent the standard error of the mean.

Workflow Complexity (Lines of Code)

Code complexity was quantified as the number of effective lines of code (LOC), defined as non-blank, non-comment statements in the workflow functions and any required helper functions used to reproduce the same set of figures and output files for each library. The resulting LOC counts are: `FastMDAnalysis`: 5 LOC; MDTraj: 103 LOC; MDAnalysis: 118 LOC.

Verification

Successful reproduction will generate all output files listed in Section 2.5 with performance characteristics matching those reported in the JCC submission (Figure 1 of the main manuscript).

References

[1] McGibbon, R.T.; Beauchamp, K.A.; Harrigan, M.P.; et al. MDTraj: A Modern Open Library for the Analysis of Molecular Dynamics Trajectories. *Biophys. J.* **2015**, *109*(8), 1528–1532. doi:10.1016/j.bpj.2015.08.015

[2] Michaud-Agrawal, N.; Denning, E.J.; Woolf, T.B.; Beckstein, O. MDAnalysis: A Toolkit for the Analysis of Molecular Dynamics Simulations. *J. Comput. Chem.* **2011**, *32*(10), 2319–2327. doi:10.1002/jcc.21787

[3] Pedregosa, F.; Varoquaux, G.; Gramfort, A.; et al. Scikit-learn: Machine Learning in Python. *J. Mach. Learn. Res.* **2011**, *12*, 2825–2830.

[4] Virtanen, P.; Gommers, R.; Oliphant, T.E.; et al. SciPy 1.0: Fundamental Algorithms for Scientific Computing in Python. *Nat. Methods* **2020**, *17*(3), 261–272. doi:10.1038/s41592-019-0686-2

[5] Baker, E.N.; Hubbard, R.E. Hydrogen Bonding in Globular Proteins. *Prog. Biophys. Mol. Biol.* **1984**, *44*(2), 97–179. doi:10.1016/0079-6107(84)90007-5

[6] Kabsch, W.; Sander, C. Dictionary of Protein Secondary Structure: Pattern Recognition of Hydrogen-Bonded and Geometrical Features. *Biopolymers* **1983**, *22*(12), 2577–2637. doi:10.1002/bip.360221211

[7] Shrake, A.; Rupley, J.A. Environment and Exposure to Solvent of Protein Atoms. Lysozyme and Insulin. *J. Mol. Biol.* **1973**, *79*(2), 351–371. doi:10.1016/0022-2836(73)90011-9

[8] Ester, M.; Kriegel, H.-P.; Sander, J.; Xu, X. A Density-Based Algorithm for Discovering Clusters in Large Spatial Databases with Noise. In *Proceedings of the 2nd International Conference on Knowledge Discovery and Data Mining (KDD-96)*; AAAI Press: Portland, Oregon, 1996; pp 226–231.

[9] van der Maaten, L.; Hinton, G. Visualizing Data Using t-SNE. *J. Mach. Learn. Res.* **2008**, *9*(86), 2579–2605.

[10] Neidigh, J.W.; Fesinmeyer, R.M.; Andersen, N.H. Designing a 20-Residue Protein. *Nat. Struct. Biol.* **2002**, *9*(6), 425–430. doi:10.1038/nsb798

[11] Aina, A. Molecular Dynamics Simulation Dataset: TrpCage Miniprotein (PDB: 1L2Y). 2025. doi:10.5281/zenodo.17755214

[12] Aina, A.; Hsueh, S.C.C.; Plotkin, S.S. PROTHON: A Local Order Parameter-Based Method for Efficient Comparison of Protein Ensembles. *J. Chem. Inf. Model.* **2023**, *63*(11), 3453–3461. doi:10.1021/acs.jcim.3c00145

[13] Aina, A.; Hsueh, S.C.C.; Plotkin, S.S. Molecular Dynamics-Generated Ensemble Dataset of Ubiquitin; for "PROTHON: A Local Order Parameter-Based Method for Efficient Comparison of Protein Ensembles". 2023. doi:10.5281/zenodo.7792287
